# Uncovering molecular features driving lung adenocarcinoma heterogeneity in patients who formerly smoked

**DOI:** 10.1186/s12967-024-05437-8

**Published:** 2024-07-08

**Authors:** Peiyao Wang, Raymond Ng, Stephen Lam, William W. Lockwood

**Affiliations:** 1 Department of Integrative Oncology, BC Cancer Research Institute, 675 West 10th Avenue, Vancouver, BC V5Z 1G1 Canada; 2 Interdisciplinary Oncology Program, Faculty of Medicine, 570 West 7th Avenue, Vancouver, BC V5Z 4S6 Canada; 3 Department of Computer Science, University of British Columbia, 2366 Main Mall, Vancouver, BC V6T 1Z4 Canada; 4 Department of Pathology and Laboratory Medicine, University of British Columbia, 899 West 12th Avenue, Vancouver, BC V5Z 4E6 Canada

**Keywords:** Lung adenocarcinoma, Smoking, Gene expression, Prognosis, Treatment selection

## Abstract

**Background:**

An increasing proportion of lung adenocarcinoma (LUAD) occurs in patients even after they have stopped smoking. Here, we aimed to determine whether tobacco smoking induced changes across LUADs from patients who formerly smoked correspond to different biological and clinical factors.

**Methods:**

Random forest models (RFs) were trained utilizing a smoking associated signature developed from differentially expressed genes between LUAD patients who had never smoked (NS) or currently smoked (CS) from TCGA (*n* = 193) and BCCA (*n* = 69) cohorts. The RFs were subsequently applied to 299 and 131 formerly smoking patients from TCGA and MSKCC cohorts, respectively. FS were RF-classified as either CS-like or NS-like and associations with patient characteristics, biological features, and clinical outcomes were determined.

**Results:**

We elucidated a 123 gene signature that robustly classified NS and CS in both RNA-seq (AUC = 0.85) and microarray (AUC = 0.92) validation test sets. The RF classified 213 patients who had formerly smoked as CS-like and 86 as NS-like from the TCGA cohort. CS-like and NS-like status in formerly smoking patients correlated poorly with patient characteristics but had substantially different biological features including tumor mutational burden, number of mutations, mutagenic signatures and immune cell populations. NS-like formerly smoking patients had 17.5 months and 18.6 months longer overall survival than CS-like patients from the TCGA and MSKCC cohorts, respectively.

**Conclusions:**

Patients who had formerly smoked with LUAD harbor heterogeneous tumor biology. These patients can be divided by smoking induced gene expression to inform prognosis and underlying biological characteristics for treatment selection.

**Supplementary Information:**

The online version contains supplementary material available at 10.1186/s12967-024-05437-8.

## Introduction

Lung cancer is the most lethal cancer in the world, with the majority of cases attributed to tobacco smoking. Successes in tobacco smoking control policies and smoking cessation programs have led to a decrease in the number of people who are actively smoking [1]. In countries such as the United States and Canada, over 50% of lung cancer deaths are now in people who had stopped smoking [2]. As a history of smoking is the biggest risk factor in terms of lung cancer development, people who have previously smoked are at an elevated risk. While lung cancer can occur even years after smoking cessation, [3] risk decreases gradually over time after smoking cessation at a rate that varies among individuals for reasons that remain unclear [4]. A recent meta-analysis showed the reducible relative risk after smoking cessation only marginally declines after 15 years from 26.7% (95% CI 20.2–34.3) to 19.7% (95% CI 13.3–26.4) at 20 years [3]. This motivates the study of tumor biology in formerly smoking lung cancer patients to determine underlying biological traits that may otherwise separate this population beyond clinical characteristics for the purposes of risk stratification.

In terms of clinical research, patients who have formerly smoked (FS) are often treated the same as those who are currently smoking, grouped together as ‘ever smokers’. However, a recent study that subdivided its cohort into patients who had never smoked (NS), patients who currently smoked (CS), or FS showed that CS have significantly greater survival after PD-L1 inhibitor treatment than patients who previously smoked in refractory NSCLC, with NS experiencing significantly worse survival compared to both groups [5] In addition, another study showed that smoking exposure can be quantified using tumor mutational burden (TMB) and transversion/transition ratio, which can be applied to classify NS, CS, FS who quit in the last 15 years and those who quit over 15 years ago [6]. This supports the idea that FS are distinct from CS as well as among themselves, although the other molecular features that separate FS and how they can translate to clinical management and treatment strategies is currently unknown.

In this study, we aimed to further understand the diversity of FS with lung adenocarcinoma (LUAD) - the most common lung cancer subtype - by exploring their tumor biology and molecular features. We hypothesized that a subset of FS patients develop cancer due to the carcinogenic effects of previous tobacco smoking, while others may develop cancer through processes unrelated to smoking. To this end, we developed an active smoking associated gene expression signature to classify LUADs from FS, which revealed distinct subsets related to either CS or NS LUADs. Furthermore, we demonstrated that these subsets have unique underlying molecular features that influence heterogeneity in tumor biology across FS. This insight towards the mechanisms underlying tumor development in people who have stopped smoking have potential implications for treatment and clinical management of the largest LUAD patient group in the future.

## Methods

### Data sources

Gene expression data from LUAD tumor samples with information regarding patient smoking status were obtained from three sources. The Cancer Genome Atlas (TCGA) dataset contained 500 RNA-Seq samples (118 CS, 75 NS, 307 FS) (Illumina HiSeq RNA -Seq V2 RSEM) that were downloaded from Broad GDAC Firehose. Somatic copy number alterations (SCNAs), mutation frequency, and other genomic data including TMB and fraction of genome altered were also obtained from available samples.

The British Columbia Cancer Agency (BCCA) dataset comprised of 69 microarray samples (39 CS, 30 NS) profiled using the Illumina WG-6 v3.0 BeadChip and the Memorial Sloan Kettering Cancer Center (MSKCC) dataset involved 192 samples (25 CS, 36 NS, 131 FS) profiled using Affymetrix HG-U133A Arrays. Both microarray datasets were obtained from the GEO database (GSE75037 and GSE31547, respectively).

### Differentially expressed Gene (DEG) analysis

Differentially expressed gene (DEG) analysis was conducted between NS and CS samples to develop a gene signature associated with active smoking. For both TCGA and BCCA datasets, genes expressed at low levels were removed and in instances of multiple probes corresponding to a single gene, only the probe with the highest mean expression was retained. Normalization was applied to each dataset using the *EdgeR* package in R and significantly up- and down-regulated genes were obtained using the *limma* package [7].

The overlapping DEGs from these two independent analyses that were also present within the MSKCC dataset constituted our active smoking gene signature. Principal component analysis (PCA) was performed using gene expression data from the genes within the signature derived from DEG analyses using the *ggfortify* package [8]. Receiver operating characteristic (ROC) curves were constructed with the principle component 1 values from each dataset’s PCA and respective areas under the curve (AUCs) were calculated to determine the ability of the gene signature to separate samples based on their NS and CS status.

### Functional analysis of DEGs

To understand the functions and pathways associated with the genes within the smoking associated gene signature, Gene Ontology (GO), [9, 10] specifically Biological Process terms, and KEGG [11] databases were used. The DAVID tool [12] allowed integration of GO terms and pathways into clusters and ShinyGO [13] was utilized for confirmatory analysis and visualization purposes.

### Random forest from gene signature

A random forest model (RF) utilizing genes of the derived gene signature and sex as features was trained to predict NS and CS status for future application to FS samples. RFs were created for both RNA-Seq and microarray data to account for inherent differences in the two data types. Each RF utilized the default settings from the *randomForest* package [14]. The RF built from RNA-Seq data was trained on 70% of the NS and CS within the TCGA dataset and tested on the remaining 30%, each of which was selected as described below. The RF built from microarray data was trained on the BCCA dataset and tested on the NS and CS of the MSKCC dataset. *YuGene* transformation was applied to both microarray datasets for cross-platform data consistency [15]. Performance metrics used to evaluate each RF included ROC curve AUC, overall accuracy, sensitivity, specificity, positive predictive value, and negative predictive value, courtesy of the *caret* package [16]. The train-test sets used for the TCGA RF were resampled 10 times and both RFs were built on 10 distinct seeds. The model with the AUC closest to the average among each dataset was selected for classification of FS.

### Random forest classification of patients who had formerly smoked

The RFs categorize samples as NS or CS based on the proportion of trees voting for either status. For FS, these classifications are interpreted as being “NS-like” or “CS-like”, respectively.

Using RF classified NS-like or CS-like status given to lung tumors of FS, the relationships between FS and different clinical and biological characteristics could be investigated. RF defined FS classes were compared to variables that have been used to delineate higher risk of lung cancer, which include individuals between the ages of 50 and 80 who have previously smoked that have quit within the last 15 years and have over 20 pack years of smoking history according to the United States Preventive Services Task Force (USPSTF) [17].

Genomic traits including TMB, fraction of genomic altered, and number of mutations were compared across samples of all smoking statuses in available data. Frequency of oncogenic driver mutations and sex were also analyzed between RF defined FS classes.

When analyzing relationships between FS class and other traits, Fisher’s exact test was used for categorical variables and Wilcoxon test was used for continuous variables. Correlations were assessed by Pearson correlation coefficient. In any comparisons that involved FS class as well as true NS and CS, Benjamini-Hochberg multiple testing correction was applied.

### Copy number assessment and mutational analysis

GISTIC 2.0 [18] was used to identify frequent SCNAs in all smoking status groups within the TCGA dataset. The parameters of q-value, confidence, and focal length were set with 0.05, 0.95, and 0.5, respectively.

A total of 220 samples had mutation data for comparison of mutational signatures between all smoking statuses in the TCGA dataset. This was analyzed using the *mutSignatures* package and comparisons were made between smoking status groups by Wilcoxon test. From the mutation data of 144 FS patients from TCGA, driver mutation frequencies of each gene were compared between NS-like and CS-like samples using Fisher’s exact test. Multiple testing correction was subsequently applied with the Benjamini-Hochberg method.

### DEG analysis in patients who had formerly smoked

As with the DEG analysis between NS and CS samples, DEGs between NS-like and CS-like FS in the TCGA dataset were identified using the *edgeR* and *limma* packages. The thresholds for DEG selection were |log2 fold change| >1 and adjusted p value < 0.01. Functional analysis on these DEGs were performed using the DAVID tool and visualized with ShinyGO.

### Immune cell content assessment

CIBERSORTx, [19] a deconvolution algorithm, was employed to estimate the relative proportion of 22 types of immune cells in the tumor tissue through the gene expression levels of 547 genes. The normalized gene expression data of the FS in the TCGA dataset were uploaded to the CIBERSORTx web interface with parameters set at 1000 permutations and relative proportions mode. Differences in each immune cell population between FS classes were compared by Wilcoxon test and adjusted by Bonferroni correction. The same process was repeated for true NS and CS as controls.

### Assessment of clinical outcomes

The pathological stage of NS-like and CS-like FS samples was evaluated by Fisher’s exact test and Benjamini-Hochberg multiple testing correction. In the TCGA dataset, FS were also assessed for their pathological T and N stages.

Univariate Cox regression analysis was conducted in the FS of the TCGA dataset using the *survivalAnalysis* package [20]. This was done to determine if RF-classified FS class could serve as an independent prognostic factor alongside age, sex, and pack year history, years since quitting.

Kaplan-Meier survival curves were constructed with FS in both TCGA and MSKCC datasets to understand differences in overall survival between NS-like and CS-like FS. The *survival* and *survminer* packages were chosen for survival analysis by log rank test and for visualization, respectively, due to their robust capabilities and compatibility with one another [21, 22].

### Statistical analysis

All statistical analyses were done using R (version 4.3.0) and p values < 0.05 were considered statistically significant. All visualization of data was conducted with the *ggplot2* and *ggpubr* packages unless otherwise stated.

## Results

### Derivation of an active smoking gene expression signature to separate patients who had never smoked and currently smoke

**Fig. 1:**
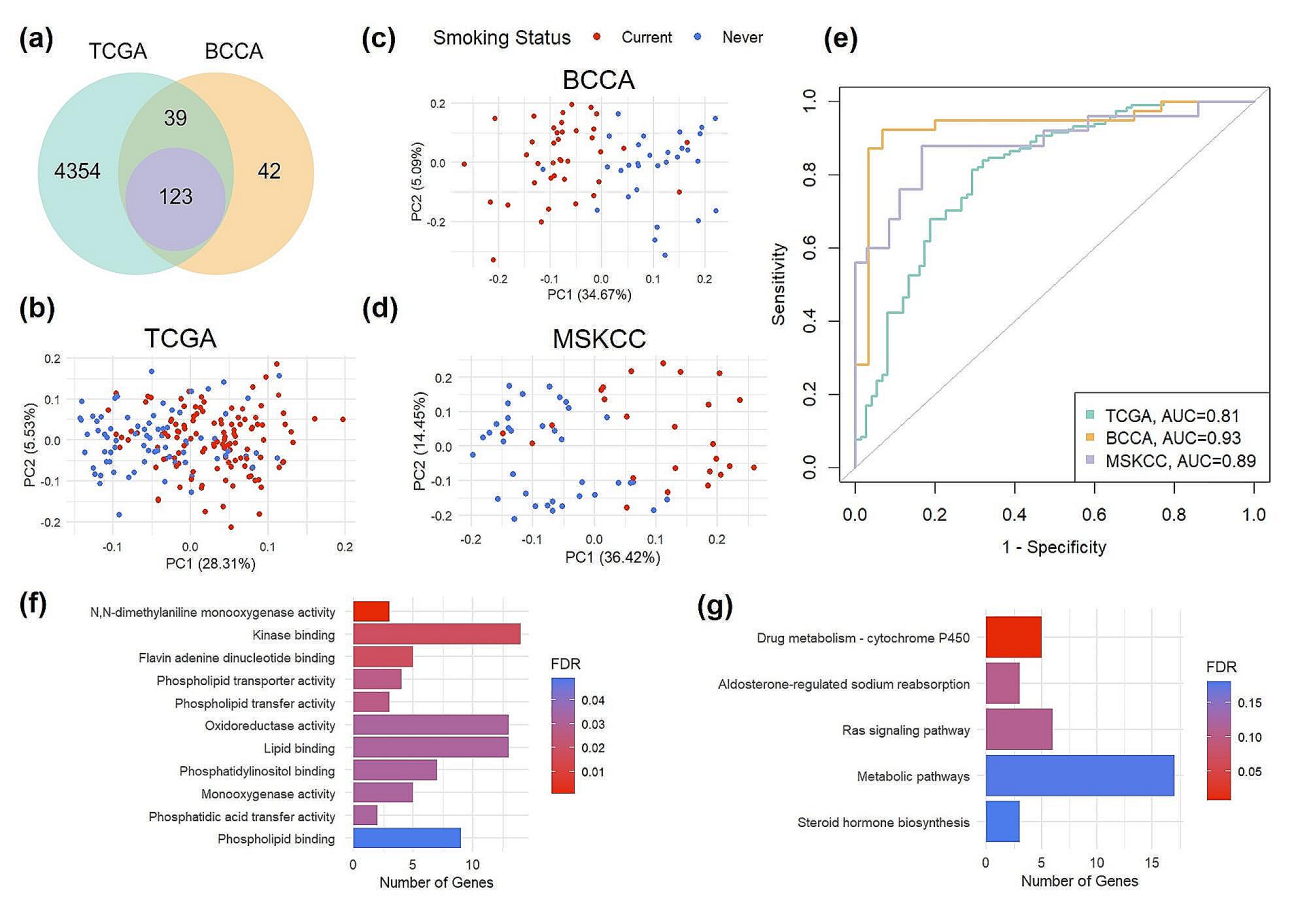
Gene signature that discerns lung adenocarcinoma patients who had never smoked (NS) and currently smoked (CS) functionally relates to drug metabolism. **(a)** Overlap of DEGs between lung adenocarcinoma tumors of NS and CS from the TCGA and BCCA cohorts that are present within the MSKCC cohort (123 genes). **(b)** PCA of expression of 123 overlapped genes in NS and CS from TCGA (*n* = 193), **(c)** BCCA (*n* = 69), and **(d)** MSKCC (*n* = 61). **(e)** ROC curves and AUCs generated from principle component 1 values for each sample, demonstrating strong separation of NS and CS by the selected 123 genes. **(f)** Functional analysis displaying significantly enriched Molecular Function Gene Ontology terms and **(g)** KEGG pathways from the 123 DEGs between NS and CS in TCGA and BCCA. FDR = false discovery rate; AUC = area under the curve

Although all FS share a history of tobacco use, the range of their smoking history and susceptibility to tobacco smoke means that lung carcinogenesis in some FS is inevitably attributable to smoking, while in others, it may be due smoking unrelated factors. We aimed to stratify the FS lung adenocarcinoma population and thus better understand their heterogeneity by building a model to classify FS based on their smoking induced gene expression. In order to determine genes related to active smoking for later classification of FS, differentially expressed gene (DEG) analysis between NS and CS in both TCGA and BCCA cohorts was conducted, which yielded 4515 and 203 DEGs, respectively. The overlap between these genes and the ones available within the MSKCC dataset resulted in a 123-gene signature (Fig. 1a). Construction of PCAs using the expression levels from these 123 genes showed a visible separation between the NS and CS patients in all datasets, including within MSKCC, which was independent from the signature derivation process (Fig. 1b-d). A receiver operating characteristic area under the curve (AUC) comprised from each PCA’s principal component 1 demonstrates that the 123-gene signature is robust in distinguishing NS and CS LUAD tumors. The AUCs for TCGA, BCCA, and MSKCC were 0.81, 0.93, and 0.89, respectively (Fig. 1e).

Functional analysis of the gene signature demonstrated that many of the 123 genes are related to regulation of monooxygenase activity (Fig. 1f). Molecular function GO terms that were most significantly enriched were “N, N-dimethylaniline monooxygenase activity”, “kinase binding”, and “flavin adenine dinucleotide binding”. The genes associated with each term and whether they are more highly expressed in CS or NS are detailed in Supplementary Table 1.

According to the KEGG pathway database, the 123 genes most commonly fell into drug metabolism by cytochrome P450, metabolic, aldosterone-regulated sodium reabsorption, and Ras signaling pathways (Fig. 1g). However, only the drug metabolism by cytochrome P450 pathway was significantly enriched (fold enrichment = 13.4, FDR = 0.007) and all the five genes that fall within this pathway (FMO3, FMO2, FMO4, MAOB, CYP3A5) are upregulated in NS tumor tissue.

**Fig. 2:**
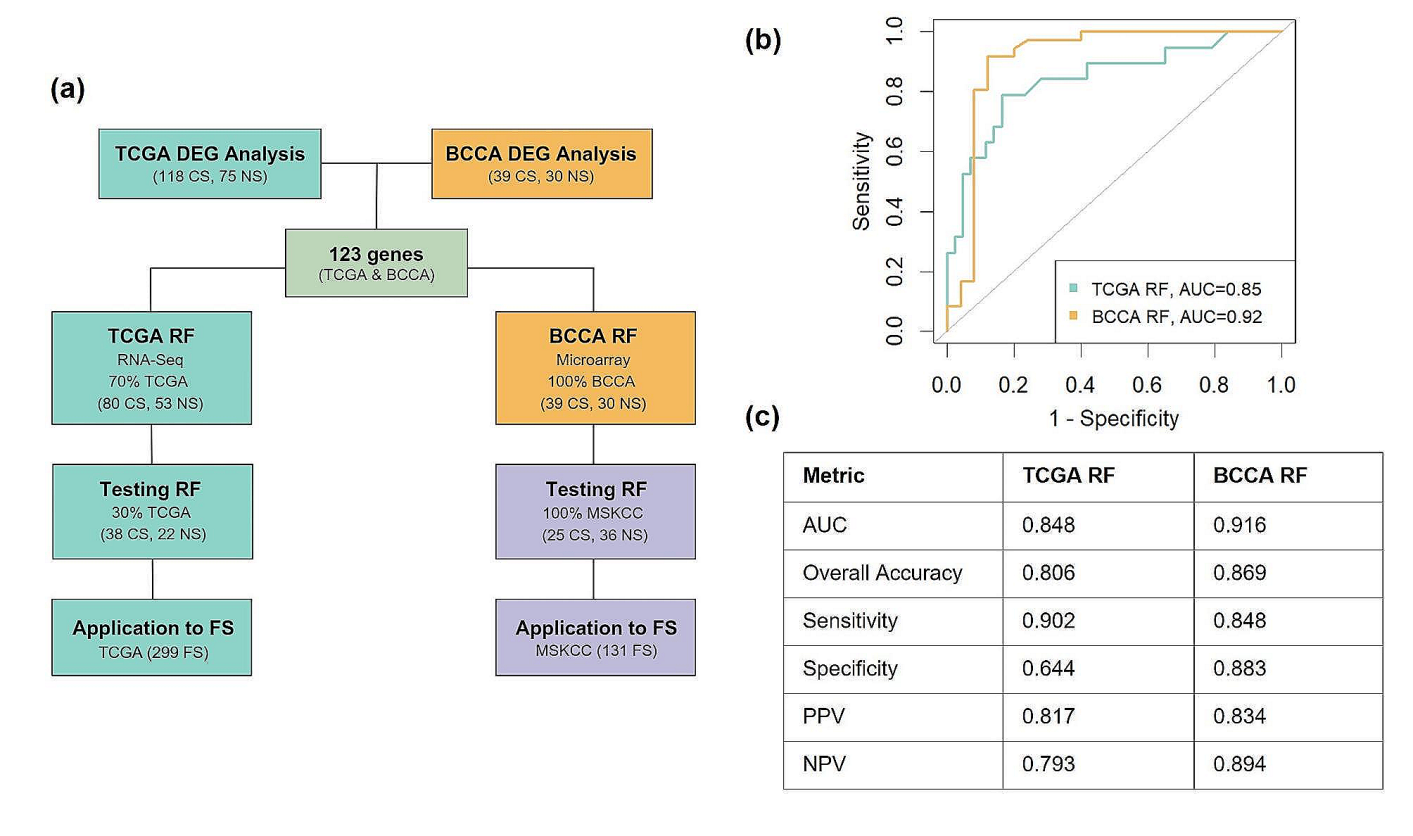
Pipeline of analysis and demonstration that random forest models (RFs) trained on gene signature and sex data can accurately distinguish between NS and CS with lung adenocarcinoma. **(a)** RF development and validation pipeline to differentiate NS and CS lung adenocarcinoma tumors. **(b)** ROC curves and AUCs generated from inputting previously unseen test data into random forest models trained on gene signature and sex data from TCGA (RNA-seq) and BCCA (microarray) datasets. **(c)** Table of random forest performance metrics. TCGA data was resampled 10 times for test and train sets and both models were built on 10 separate seeds and mean metrics are shown. AUC = area under the curve, PPV = positive predictive value, NPV = negative predictive value

Random forest models (RFs) were built to classify NS and CS LUAD patients with both RNA-seq data and microarray data using these 123 genes and sex as input features (Fig. 2a). These models were then validated by predicting NS and CS status from independent test data; the RNA-seq RF was trained on 70% of the TCGA dataset (*n* = 133) and tested on the remaining 30% (*n* = 60). The microarray RF was trained by the BCCA cohort (*n* = 69) and tested on the MSKCC data (*n* = 61). The AUCs from the validation these models were 0.85 and 0.92, respectively (Fig. 2b), with further performance metrics listed in the table of Fig. 2c.

### Smoking induced gene expression correlates modestly with patient characteristics

**Fig. 3:**
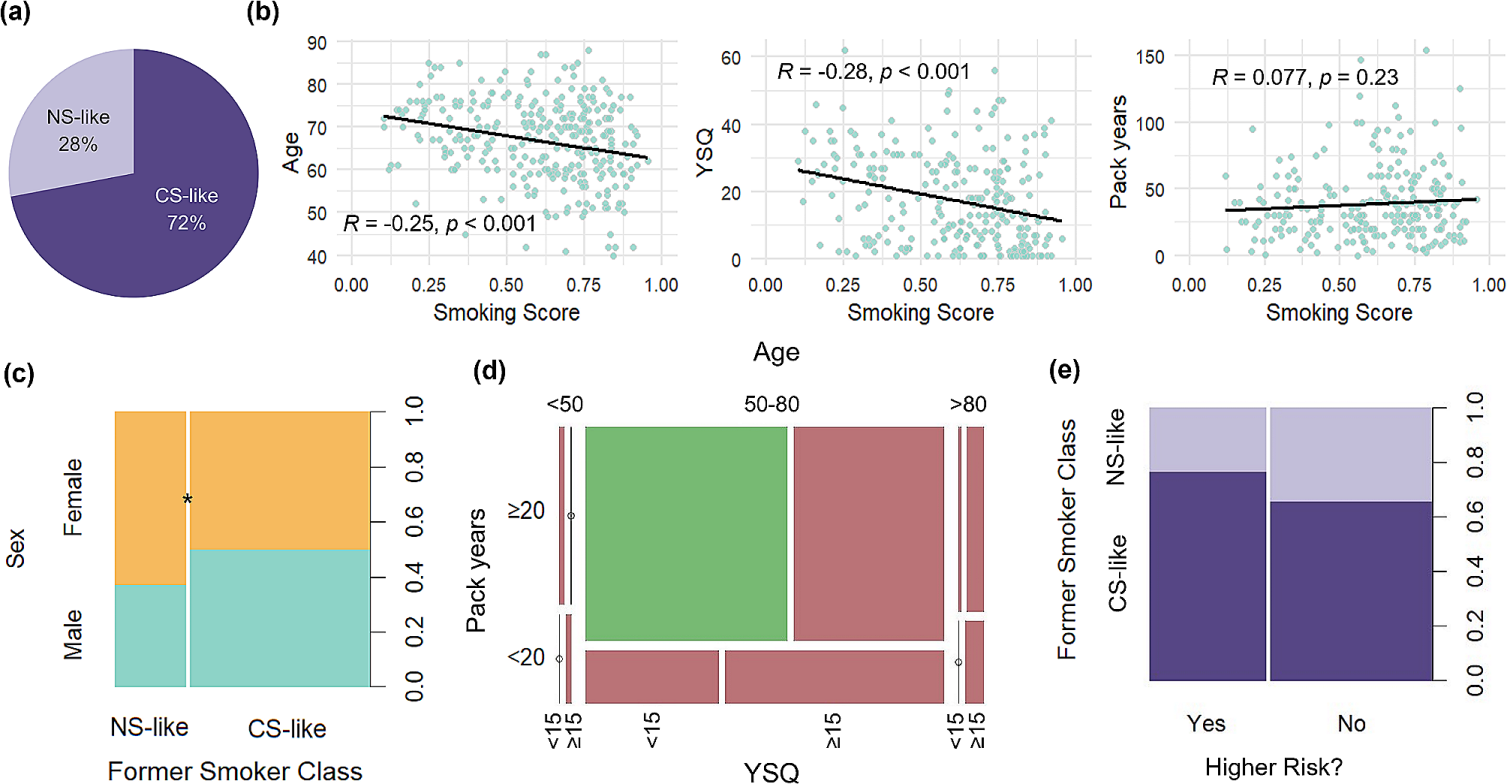
Clinical characteristics correlate mildly with smoking induced gene expression and patients who formerly smoked (FS) with lung adenocarcinoma are a diverse demographic. **(a)** Percentage of FS categorized as either NS-like (*n* = 86) or CS-like (*n* = 213) by random forest model (RF). **(b)** Age, years since quitting (YSQ), and pack years depending on smoking score as predicted by RF. **(c)** Proportions of sex in FS relative to their RF classified CS- or NS-like status. **(d)** Mosaic plot of FS who would be higher risk and thus more likely recommended for screening (green) or lower risk (red). **(e)** Proportion of RF classified NS- or CS-like FS that are high or low risk. *p < 0.05

The patients who had previously smoked from the TCGA cohort were defined as 72% (*n* = 213) CS-like and 28% (*n* = 86) NS-like according to our RF (Fig. 3a). The RF classifies a patient as NS-like or CS-like on a scale from zero to one, with a score less than 0.5 being NS-like and a score greater than 0.5 being CS-like. Correlative analyses showed that age and years since quitting have a significant but weak negative correlation with smoking score (Fig. 3b). The former is likely due to the fact that with higher age, there is a greater amount of time for years since quitting to accrue; as such, age and years since quitting are correlated with one another (data not shown). FS in this cohort were defined as those who had quit for over a year; to assess the change in proportion of NS-like FS, this group was further parsed into those who had quit more than five, 10, or 15 years. The percentage of NS-like FS increased from 29.5 to 31.4%, 34.0%, and 39.1%, respectively, although none of these proportions are significantly different. This aligns with previous findings that some smoking related genes decrease in expression linearly as time since quitting increases, while other genes’ expression remain resiliently expressed for years, potentially explaining why our NS-like status, which is defined by gene expression, is only modestly correlated with years since quitting [23]. Surprisingly, there was no correlation between smoking score in the FS and pack years (Fig. 3b). Finally, although sex is not currently part of lung cancer screening criteria, it was found that a slightly higher proportion of female FS patients were classified as NS-like compared to males (Fig. 3c).

The RF was also applied to the MSKCC dataset, which classified 57 FS to be CS-like and 74 to be NS-like. Although there was not enough information to assess the theoretical risk of these patients, available data indicated that pack years had a slight positive correlation with CS-like status and recapitulated that a greater proportion of female FS are NS-like compared to males (Figure S1a-b). Together, this suggests that clinical factors are not strongly correlated with active smoking gene expression levels in FS.

To understand how high risk traits relate to FS based on their active smoking gene expression levels, FS LUAD patients were presented to the 123-gene RF to be classified as never smoker-like (NS-like) or current smoker-like (CS-like). Only 41.7% of FS with LUAD would have been considered to be at relatively higher risk for lung cancer according to the USPSTF (Supplementary Table 2). Furthermore, of the FS who are at lower risk, 38.3% (79/206) were still categorized as CS-like (Fig. 3d-e). This demonstrates that, according to our model, a sizeable number of FS falling outside of high lung cancer risk attributes harbored tumors with high smoking induced gene expression.

### NS-like and CS-like formerly smoking patient subgroups have significantly different genomic profiles

**Fig. 4:**
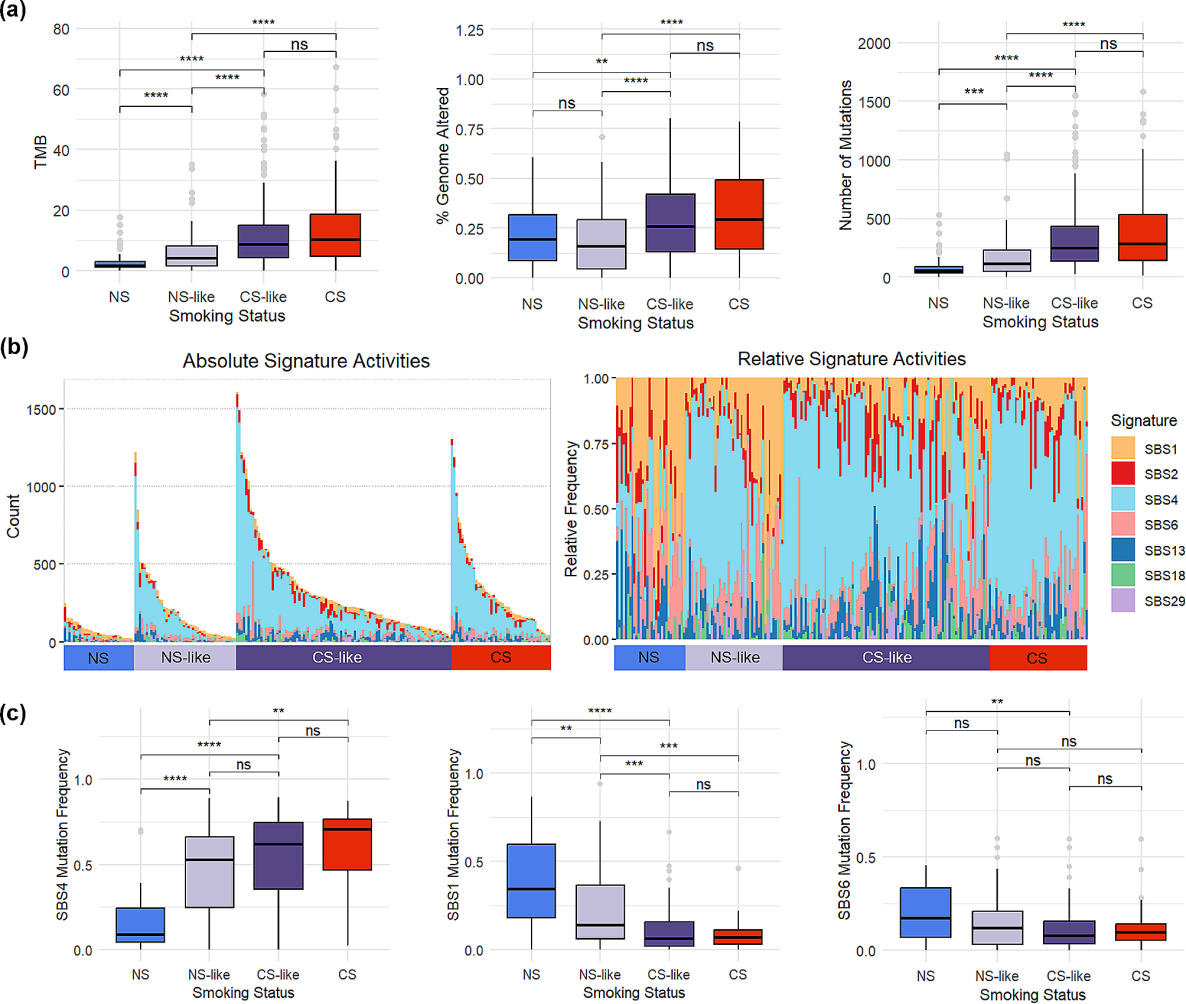
Genomic profiles between NS- and CS-like FS with lung adenocarcinoma are significantly different. **(a)** Genome related measures and **(b)** absolute and relative frequencies of mutational signatures that have been previously detected in lung cancer between true NS, true CS, and RF classified NS- and CS-like FS. **(c)** Relative levels of SBS4 (tobacco), SBS1 (ageing) and SBS6 (DNA mismatch repair) mutational signatures between different smoking statuses. TMB = tumor mutational burden, ns = not significant, *p < 0.05, **p < 0.001, ***p < 0.0001, ****p < 0.00001

In order to determine whether other underlying biological differences are associated with active smoking gene expression levels, RF-classified FS were compared to different biological features. CS-like FS have markedly higher TMB than NS-like FS and their TMB is relatively similar to those of true CS. It is notable that relative to true NS, NS-like FS have significantly greater TMB. A similar stepwise trend from NS to NS-like FS to CS-like FS is also present in the number of mutations between groups. Regarding the fraction of the genome that is altered in each group, there is no significant difference between NS and NS-like tumors. However, both of these groups have distinguishable differences compared to both CS-like and CS tumors, which again demonstrate distinct genomic differences between NS-like and CS-like FS (Fig. 4a). Combining these three genomic traits through a composite score, having quit smoking for over 15 years was also able to separate FS by genomic features in addition to RF predicted class of FS (Figure S2). Taken together, these findings extend beyond the established knowledge that NS and CS possess pronounced genomic characteristics (Figure S3). Moreover, this reveals that FS occupy an intermediate position between these two groups that can be further delineated into two distinct groups based on our active smoking gene expression signature.

Mutational signature analysis revealed different mutational signature profiles between NS-like and CS-like FS with LUAD (Fig. 4b). Specifically, relative level of SBS4, a well-defined COSMIC signature associated with tobacco mutagens, [24] is relatively higher in CS-like than NS-like FS (Figure S4) although the difference is only upon comparing absolute count of SBS4 between the two groups (Fig. 4c). This aids to verify the gene signature, which was developed to separate FS by smoking related gene expression. Furthermore, APOBEC signature SBS2 was relatively higher in NS than other groups as has been previously reported (Figure S4). True NS have the highest relative levels of SBS1 and SBS6, representing ageing and deficient mismatch repair, [24] which were significantly greater than CS-like FS but not NS-like FS, suggesting a greater impact of endogenous signatures in tumors of NS and NS-like FS (Fig. 4c).

The frequency of main oncogenic driver alterations was not significantly different between NS-like and CS-like tumors. The proportions of KRAS, EGFR, and ALK alterations were relatively similar among FS in TCGA and proportions of KRAS and TP53 alterations were also not significantly different between FS groups in MSKCC (Figure S5). Although the proportion of those with EGFR mutation was slightly higher in NS-like FS than that of CS-like FS, the significance of this difference does not hold after multiple testing correction.

**Fig. 5:**
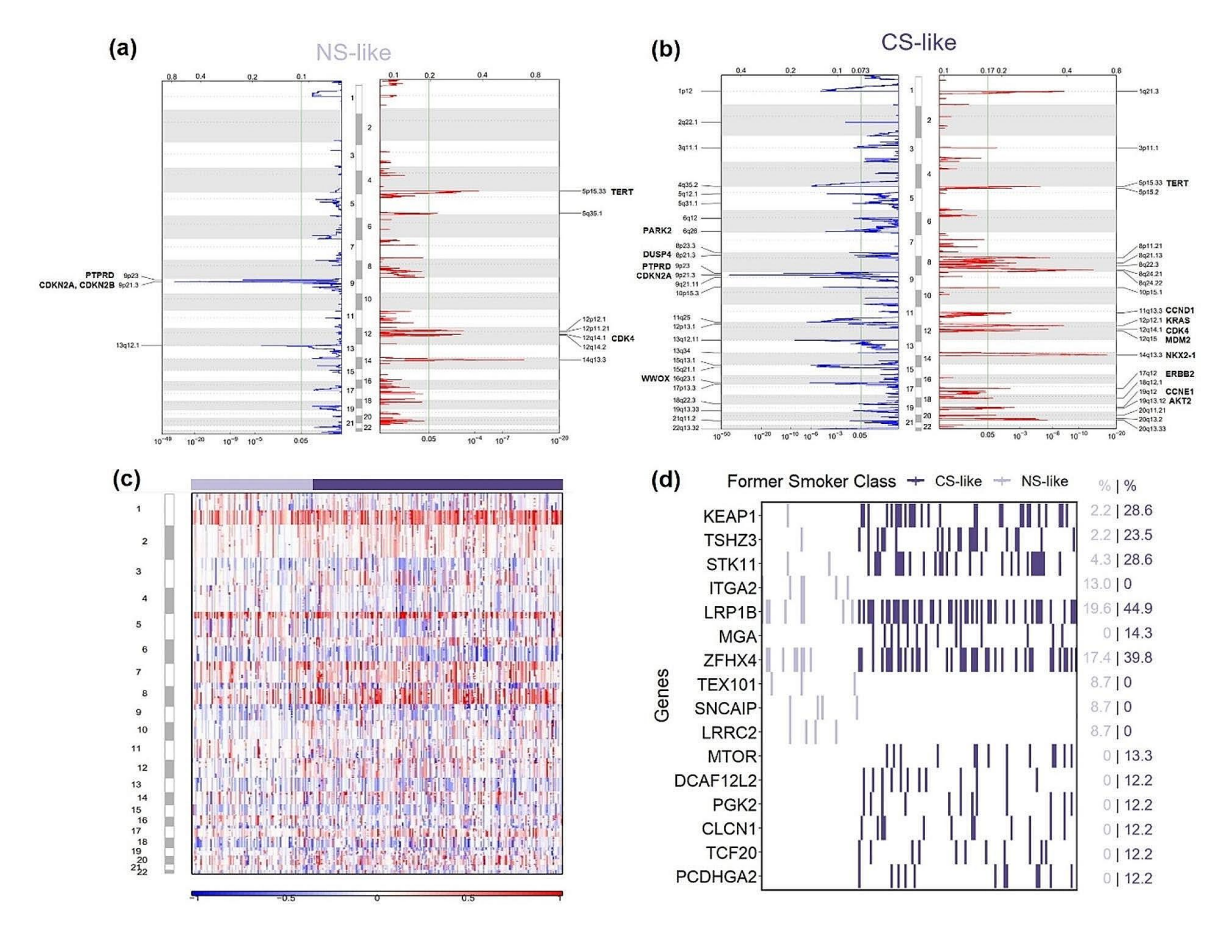
Genomic analyses show greater somatic copy number alterations in CS-like than NS-like FS with lung adenocarcinoma, and genes with significantly different mutation frequencies between FS from TCGA are shown. GISTIC 2.0 analysis of copy number amplifications (red) and deletions (blue) of **(a)** NS-like (*n* = 85) and **(b)** CS-like (*n* = 213) FS with lung adenocarcinoma patients within TCGA. Significance is delineated by a green line at 0.05 representing the false discovery rate corrected p-value. The corresponding chromosome regions are labeled, and genes of interest are indicated. **(c)** Somatic copy number profiles of all FS in TCGA. Copy number amplifications (red) and losses (blue) are plotted as a heatmap with samples on the x-axis and chromosomes on the y-axis. **(d)** Genes with significantly different mutation frequencies between NS-like and CS-like FS in TCGA (Fisher’s exact test, *n* = 144, *p* < 0.01)

SCNAs were reported in the TCGA dataset, which demonstrated that CS-like FS exhibit far more frequently altered regions of amplification and deletion compared to NS-like patients (Fig. 5a-b). In addition, compared to NS-like FS tumors, CS-like tumors demonstrated greater relative copy number alterations in multiple regions across the genome (Fig. 5c). This recapitulates the trend found in percentage of the genome altered between the two groups of FS. Regions that were significantly amplified in CS-like tumors held many genes known to be associated with cancer development, including KRAS, CDK4, and TERT. Comparing FS to true NS and CS, CS have comparable SCNAs to CS-like FS. However, NS-like FS have the least somatic copy number deletions and amplifications, even compared to true NS (Figure S6).

Significantly different mutation frequency between NS-like and CS-like FS was identified in 80 genes (*p* < 0.05), with the top 16 genes (*p* < 0.01) shown in Fig. 5d. CS-like tumors had significantly more highly mutated genes than NS-like samples, with the most significant genes being KEAP1, TSHZ3, and STK11. Genes that were significantly more frequently mutated in NS-like samples were ITGA2, TEX101, SNCAIP, and LRRC2, which had mutation frequencies ranging from 8.7 to 13.0% compared to 0% in CS-like samples (Fig. 5d). Although multiple testing adjustment did not retain any significant genes, the exploratory nature of this analysis highlights biological differences between subgroups of FS.

### Tumors of CS-like patients NS-like tumors have different transcriptional and immune profiles

**Fig. 6:**
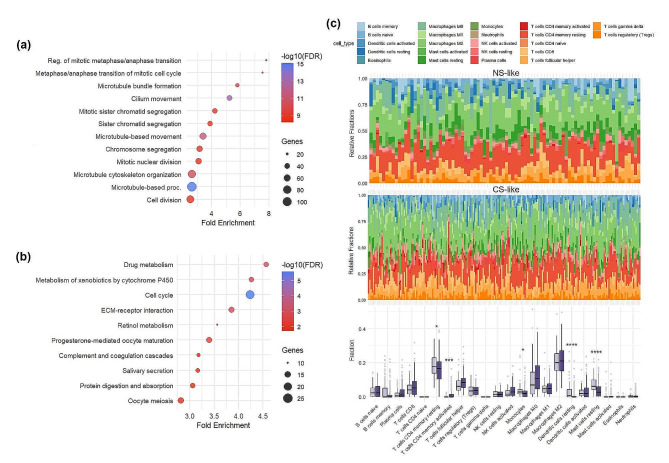
Gene expression differences between NS-like and CS-like FS with lung adenocarcinoma revolve functionally around the cell cycle and drug metabolism and myeloid lineage immune cells are more abundant in NS-like FS. **(a)** Functional analysis displaying enriched Biological Process Gene Ontology terms and **(b)** enriched KEGG pathways from the 1050 DEGs between NS-like and CS-like FS in TCGA. **(c)** Relative fraction of 22 immune cell types in NS-like (*n* = 86) and CS-like (*n* = 213) FS in TCGA and comparisons of tumor-infiltrating immune cells between NS-like and CS-like FS (Wilcoxon test and Bonferroni correction, *n* = 193, *p < 0.05, ****p < 0.0001). FDR = false discovery rate

Upon investigating transcriptomic differences between NS-like and CS-like FS, the majority of significantly enriched GO terms relate to the cell cycle (Fig. 6a). This aligns with KEGG pathway analysis indicating the cell cycle as the most significantly enriched pathway, followed by drug metabolism, metabolism of xenobiotics, and ECM-receptor interaction (Fig. 6b).

Tumor immune microenvironment was investigated within FS using CIBERSORTx, which revealed that heterogeneity exists within NS-like and CS-like FS (Fig. 6c). In addition, differences between these subgroups of FS exist in that CS-like FS have greater proportions of activated CD4 memory T cells while NS-like FS have greater proportions of resting CD4 memory T cells, monocytes, dendritic cells, and mast cells after Bonferroni correction (Fig. 6c). Comparing true CS to NS, it is observed that myeloid lineage cells, specifically dendritic cells and mast cells, are present in higher proportions among NS. Conversely, lymphoid lineage plasma cells and T follicular helper cells levels are proportionally greater in CS (Figure S7).

### CS-like patients who had formerly smoked tend to have LUAD in later pathological stage and worse overall survival

**Fig. 7:**
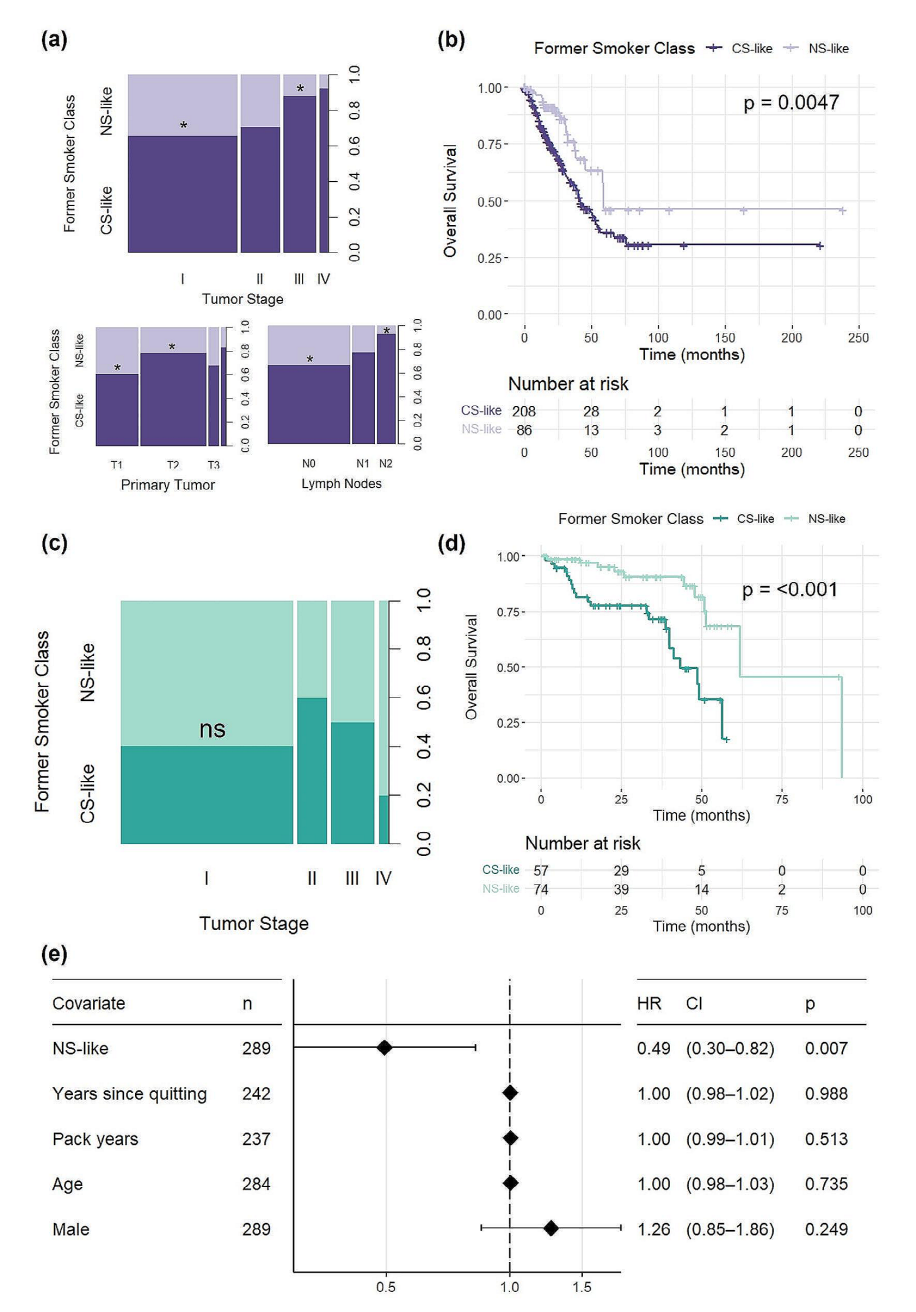
CS-like FS with lung adenocarcinoma in TCGA have more advanced tumors and their overall survival is significantly worse than NS-like FS in both TCGA and MSKCC. Proportion of RF classified NS- and CS-like FS in different tumor stages and classifications in the **(a)** TCGA and **(c)** MSKCC cohort. **(b)** Kaplan Meier survival curve and number at risk table showing overall survival between NS- and CS-like FS. Median survival difference is 17.5 months in the TCGA cohort and **(d)** median survival difference is 18.6 months in the MSKCC cohort. **(e)** Univariate Cox regression of overall survival depending on RF-classified status in FS and other clinical characteristics in the TCGA cohort

To assess how active smoking gene expression is related to tumor characteristics and clinical outcomes, RF-classified FS were then associated with variables related to tumor stage and overall survival. In the TCGA dataset, CS-like patients more frequently present with tumors at advanced pathological stages compared to NS-like patients. After multiple testing adjustment, CS-like FS patients are significantly more likely to have stage III tumors compared to NS-like patients, whereas those who are NS-like have a higher likelihood of being in stage I than CS-like patients (Fig. 7a). The TNM staging data further illustrate that CS-like patients have a trend towards larger tumor size and increased lymph node involvement compared to NS-like patients. Notably, this difference in proportion is significant between T1 and T2 tumors as well as N0 and N2 tumors even after multiple testing correction. There were not enough metastatic events in the formerly smoking patient cohort to test for differences in proportion in metastasis (Fig. 7a).

Kaplan-Meier survival analysis of NS-like and CS-like FS followed by log rank test for statistical significance established that overall survival differs between the two subgroups of FS. Prognosis is 17.5 months longer in NS-like FS than CS-like FS, with a median survival time of 59.1 months compared to 41.6 months (Fig. 7b). This is consistent with CS-like patients demonstrating higher pathological stages of LUAD, which directly correlates to poorer survival outcomes (data not shown). In the MSKCC dataset, there is no significant difference in pathological stage between NS-like and CS-like groups (Fig. 7c). However, similarly to the TCGA dataset, NS-like FS have significantly longer overall survival than CS-like FS, with a median survival difference of 18.6 months (Fig. 7d). Importantly, other univariate survival analyses conducted demonstrated that NS-like FS are 49% less likely to die than CS-like FS (HR = 0.49, CI = 0.29, 0.81) and no other established clinical variables significantly affect overall survival in FS, including years since quitting, pack years, age, and sex (Fig. 7e). This suggests that our active smoking classifier is a more effective independent prognostic factor than any of these aforementioned clinical variables.

## Discussion

A portion of the heterogeneity in the tumor biology of FS is due to the varied influence of smoking on lung carcinogenesis. We demonstrate that the FS population can be meaningfully segregated by their smoking related gene expression. Our study allowed FS to be classified as CS-like or NS-like based on the expression of a 123 gene signature that is associated with active smoking, defined through assessment of true NS and CS LUAD tumors.

Our work shows that despite lack of correlation with clinical characteristics, smoking related gene expression has relevance in predicting other aspects of LUAD tumor biology as well as overall survival in FS. The CS-like FS class had significantly greater genomic disturbances than NS-like FS even though it did not significantly correlate with smoking pack year history or years since quitting. RF-predicted FS classes also showed a divide in mutational signature profiles, where CS-like FS had relatively greater levels of tobacco mutagen signature SBS4 and NS-like FS had relatively higher levels of endogenous signatures SBS1, SBS6, and SBS2. These trends have been previously reported in true CS and NS patients with LUAD, respectively, supporting a clear and biologically relevant subdivision within FS [25].

Higher TMB is associated with greater sensitivity to immunotherapy in NSCLC [26]. Although FS have been shown to have significantly poorer response to immunotherapy than CS, [5] it is possible that a subset of FS identifiable as CS-like FS may confer great benefit since they have comparable TMB, fraction of genome altered, mutation counts, and copy number alterations to CS. This supports previous findings that FS are separable by TMB based on years since quitting, indicating distinct biological subgroups within the FS population [6]. In addition, tumors of CS-like FS have significantly higher proportions of activated CD4 memory T cells, and high levels of tumor infiltrating lymphocytes are well documented to predict good response to PD-1 blockade [27]. A caveat is that CS-like FS harbor significantly higher mutation frequencies in KEAP1 and STK11, both of which are associated with poor response to immunotherapy even with high TMB [28, 29]. This further refines the subgroup that may exist within FS who would benefit from immunotherapy and warrants further exploration in responses to this treatment specifically in patients who had previously smoked.

Aside from differing immune profiles and genomic characteristics, DEGs between FS classes were functionally related to the cell cycle, whose dysregulation is a hallmark feature of cancer and has been observed to be more highly disrupted in true CS than NS [30]. Another highly enriched pathway from the DEGs between FS groups is metabolism of xenobiotics; genes from this pathway are more highly upregulated in NS-like than CS-like FS. Impairment in the metabolism of foreign compounds aligns with the idea that FS are more CS-like if their lung biology is less adept to process the compounds in tobacco smoke. Genes related to toxin removal from the metabolism of xenobiotics pathway including FMO3 [31] and CYP3A5 [32] are all down-regulated in CS-like tumors, potentially contributing to development of smoking induced tumors.

Although CS-like FS had significantly greater SCNAs as well as more chromosomal regions altered that belong to known tumor suppressors, some of these regions were also shared with NS-like FS, including CDKN2A. The overlap of these regions may suggest that these genes are consistently involved with LUAD of FS. Conversely, the abundance of other canonical cancer-related genes in CS-like FS may suggest distinct routes to tumorigenesis compared to NS-like tumors.

The subgroups of FS were also associated with tumor stage and overall survival. CS are more likely to be detected in advanced stage disease than FS, [33] and our findings follow this in that CS-like FS harbor a significantly higher proportion of late stage tumors than NS-like FS in the TCGA cohort. This may be a contributing factor to NS-like FS having a 17.5 month longer overall survival. However, it should be noted that there was no correlation between stage and FS classification in the MSKCC cohort, although it was also found that NS-like FS had significantly longer overall survival. In addition, no other clinical variable in univariate analyses was able to predict overall survival the way that the RF-classified FS class could. This may suggest that there are properties of certain tumors that are driven by smoking related gene expression that accelerate tumor progression, and that our gene signature may be able to identify these higher risk patients as candidates for more aggressive treatment regimens. The increased overall survival in NS-like FS is further supported by higher proportions of resting CD4 T cells, monocytes, resting dendritic cells, and resting mast cells, all of which have been previously significantly correlated with higher overall survival in LUAD patients of all smoking statuses [34]. Thus, this gene signature may be able to predict prognosis in LUAD FS patients and is a step towards personalized medicine for FS.

In our study probing tumors of LUAD FS by smoking related gene expression, there is no significant association between CS-like FS and patients who would have been considered at higher risk of developing lung cancer due to their age, pack year history, and years since quitting. Lung cancer risk over time is unique to the individual as some FS have been shown to remain at elevated risk despite quitting for more than 25 years [35]. Our work shows that CS-like tumors can present in FS who have quit for a long time, suggesting that persistently modified gene expression or genomic alterations may be a possible explanation for persistently high risk in some FS. This supports previous studies that have proposed a personalized approach to determining risk as being optimal for FS [4, 35] and research on FS should continue to investigate refinements towards early detection, including understanding genomic features of normal tissue in high risk populations.

### Limitations

A limitation of this study is the lack of public databases separating current from FS with detailed smoking history, such as pack years and years since quitting. A previous retrospective study on lung cancer screening eligibility found that 36% of patients did not have smoking history documented in their medical records system among nearly 500 patients assessed [36]. This translated to a limited sample size in our study and restricted the ability to bridge tumor transcriptomics and genomics with clinical characteristics of FS from several public datasets. Considering the heterogeneity within a tumor and the small part extracted for transcriptomic and genomic sequencing, not only a larger sample size but a standardized protocol for extracting tumor samples could be established in the future for more generalizable results. Another future direction would be to integrate methylation data in the analyses to determine if it contributes to smoking related gene expression, but this data is not yet available. A further limitation is that all cohorts utilized in this study originated from North American centers and patients were primarily Caucasian. This calls for further investigation of FS with detailed smoking history in other geographical areas with more diverse racial backgrounds to understand if the results from this study are location- or race-specific.

There was also a lack of normal tissue and longitudinal datasets, which limit the direct applicability of these findings for screening and early detection purposes. Instead, this work is able to indirectly show that the use of clinical characteristics in screening may not adequately capture people who are at the highest risk of aggressive smoking related cancer. Our study serves as a proof of concept of the heterogeneity within tumor biology of FS that can be leveraged in patient care. Future directions include replicating this analysis in FS with more diverse geographical and racial backgrounds as well as in healthy patients longitudinally to determine the dynamics of smoking related gene expression over time.

## Conclusions

FS are a diverse population not only due to variability of pack years and years since quitting, but due to differences in tumor biology. This study demonstrated the ability to stratify FS by smoking related gene expression that had weak correlations with clinical characteristics and smoking history, but was associated with underlying factors including genomic alterations, immune infiltration and clinical factors including overall survival. This demonstrates the potential of considering gene expression in the clinical care of FS as well as motivates future research that focuses on FS with lung cancer to offer them personalized care as this population continues to grow.

### Electronic supplementary material

Below is the link to the electronic supplementary material.

Supplementary Material 1

Supplementary Material 2

## Data Availability

The data that support the findings of this study are openly available in the Gene Expression Omnibus (GEO) database (https://www.ncbi.nlm.nih.gov/gds/) at GSE75037 and GSE3147 as well as in Broad GDAC Firehose (https://gdac.broadinstitute.org/) under ‘Lung adenocarcinoma’.
