# Supplementary material for: Uncovering molecular features driving lung adenocarcinoma heterogeneity in patients who formerly smoked
Source: J Transl Med. 2024 Jul 8;22:634. doi: 10.1186/s12967-024-05437-8 (PMC11229340; doi:10.1186/s12967-024-05437-8)
Supplement: Supplementary file 1 — Supplementary Material 1 [file 12967_2024_5437_MOESM1_ESM.docx]

**Supplementary**

**Supplemental Table 1: Gene Ontology Enrichment Analysis for 123 DEGs between patients who had never smoked and currently smoke from TCGA and BCCA.**

| Enrichment FDR | nGenes | Pathway Genes | Fold Enrichment | Pathway | Genes |
| --- | --- | --- | --- | --- | --- |
| 0.000819755 | 3 | 5 | 111.20 | N,N-dimethylaniline monooxygenase activity | FMO3 FMO4 FMO2 |
| 0.032113139 | 2 | 7 | 52.95 | Phosphatidic acid transfer activity | PRELID3B PRELID3A |
| 0.027044537 | 3 | 23 | 24.17 | Phospholipid transfer activity | PRELID3B PITPNM1 PRELID3A |
| 0.027044537 | 4 | 54 | 13.73 | Phospholipid transporter activity | ATP8B1 PRELID3B PITPNM1 PRELID3A |
| 0.017849716 | 5 | 84 | 11.03 | Flavin adenine dinucleotide binding | FMO3 MAOB FMO4 FMO2 TXNRD1 |
| 0.032113139 | 5 | 121 | 7.66 | Monooxygenase activity | FMO3 CYP24A1 FMO4 FMO2 CYP3A5 |
| 0.032113139 | 7 | 262 | 4.95 | Phosphatidylinositol binding | GSDMB SLC9A1 PITPNM1 RPS6KC1 IQGAP2 STXBP6 MTM1 |
| 0.049084756 | 9 | 468 | 3.56 | Phospholipid binding | GSDMB ATP8B1 SLC9A1 PITPNM1 RPS6KC1 IQGAP2 STXBP6 MTM1 GAS6 |
| 0.017849716 | 14 | 794 | 3.27 | Kinase binding | TOLLIP TPX2 PIK3IP1 PITPNM1 WWC1 KIF14 DUSP4 SOX9 TRAF2 PARP1 PIK3R1 CKS1B GAS6 CEBPA |
| 0.032113139 | 13 | 811 | 2.97 | Lipid binding | SIDT1 GSDMB ATP8B1 SLC9A1 PITPNM1 TRAF2 RPS6KC1 IQGAP2 NR3C2 STXBP6 MTM1 HSD11B2 GAS6 |
| 0.032113139 | 13 | 835 | 2.89 | Oxidoreductase activity | FMO3 CYP24A1 MAOB FMO4 SESN1 HSD17B2 FMO2 CYP3A5 FDX1 PTGES DHRS3 HSD11B2 TXNRD1 |

**Supplemental Table 2: Distribution of patients who had formerly smoked with lung adenocarcinoma in TCGA (n=206) according to United States Preventive Services Task Force.**

| n=214 |  | **Years since quitting** | |
| --- | --- | --- | --- |
| **Age** | **Pack years** | <15 | ≥15 |
| <50 | <20 | 0 | 2 |
|  | ≥20 | 4 | 0 |
| 50-80 | <20 | 14 | 23 |
|  | ≥20 | 86 | 64 |
| >80 | <20 | 0 | 4 |
|  | ≥20 | 1 | 8 |


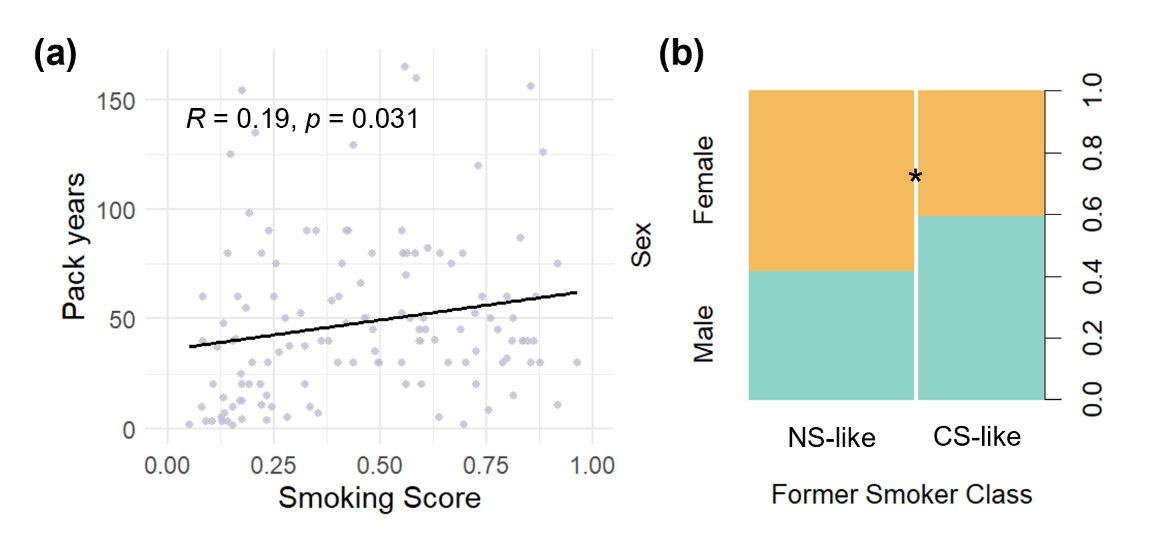


**Figure S1. Random forest (RF) defined classes of patients who formerly smoked (FS) weakly correlate pack years and sex in the FS with lung adenocarcinoma from the MSKCC cohort (n=131).** *p < 0.05


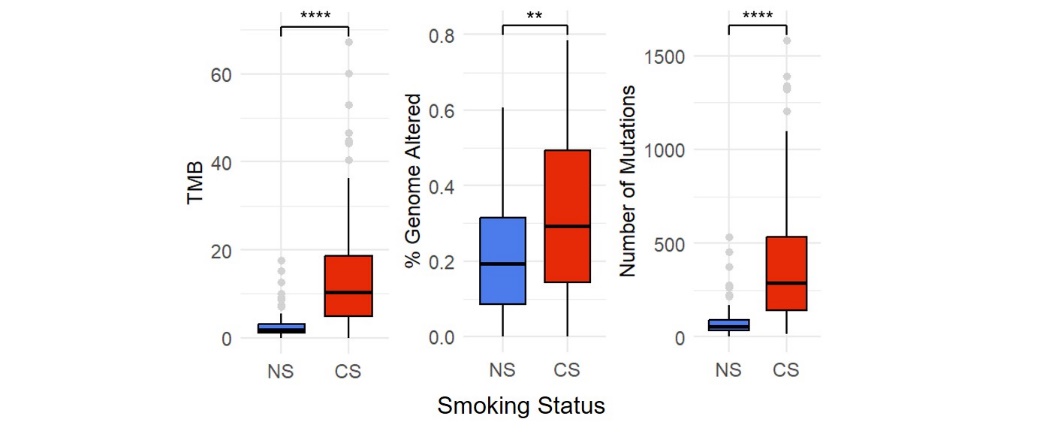


**Figure S2: Genome related measures (tumor mutational burden, fraction of genome altered, and number of mutations) are significantly different between patients who had never smoked (NS) and currently smoke (CS) with lung adenocarcinoma.** **p < 0.01, ****p < 0.0001


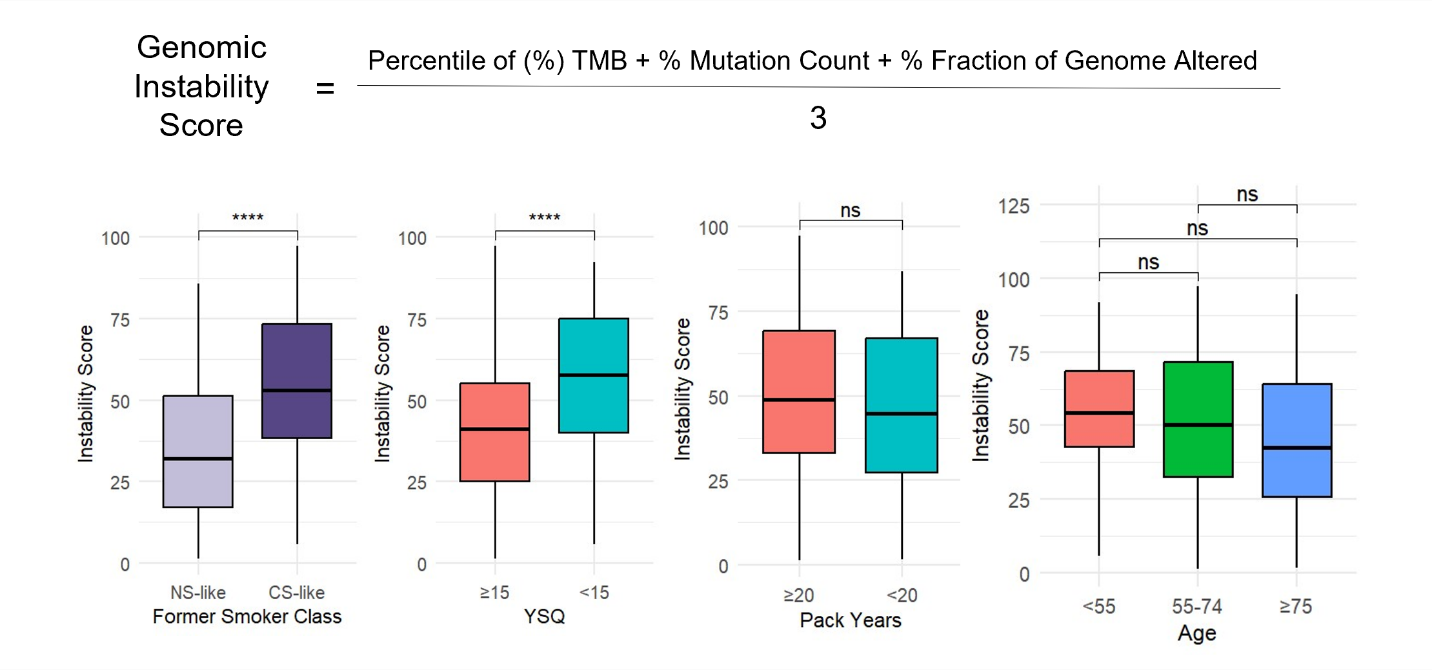


**Figure S3: Overall genomic instability can be distinguished in patients who had formerly smoked (FS) by RF-predicted class in FS and years since quitting (YSQ) at 15 years.** There are no significant differences in genomic instability of FS based on pack years and age at cut-offs for higher risk populations. Genomic instability is scored as an average percentile score of tumor mutational burden (TMB), mutation count, and fraction of genome altered in each patient who had previously smoked. ns = not significant, ****p < 0.0001


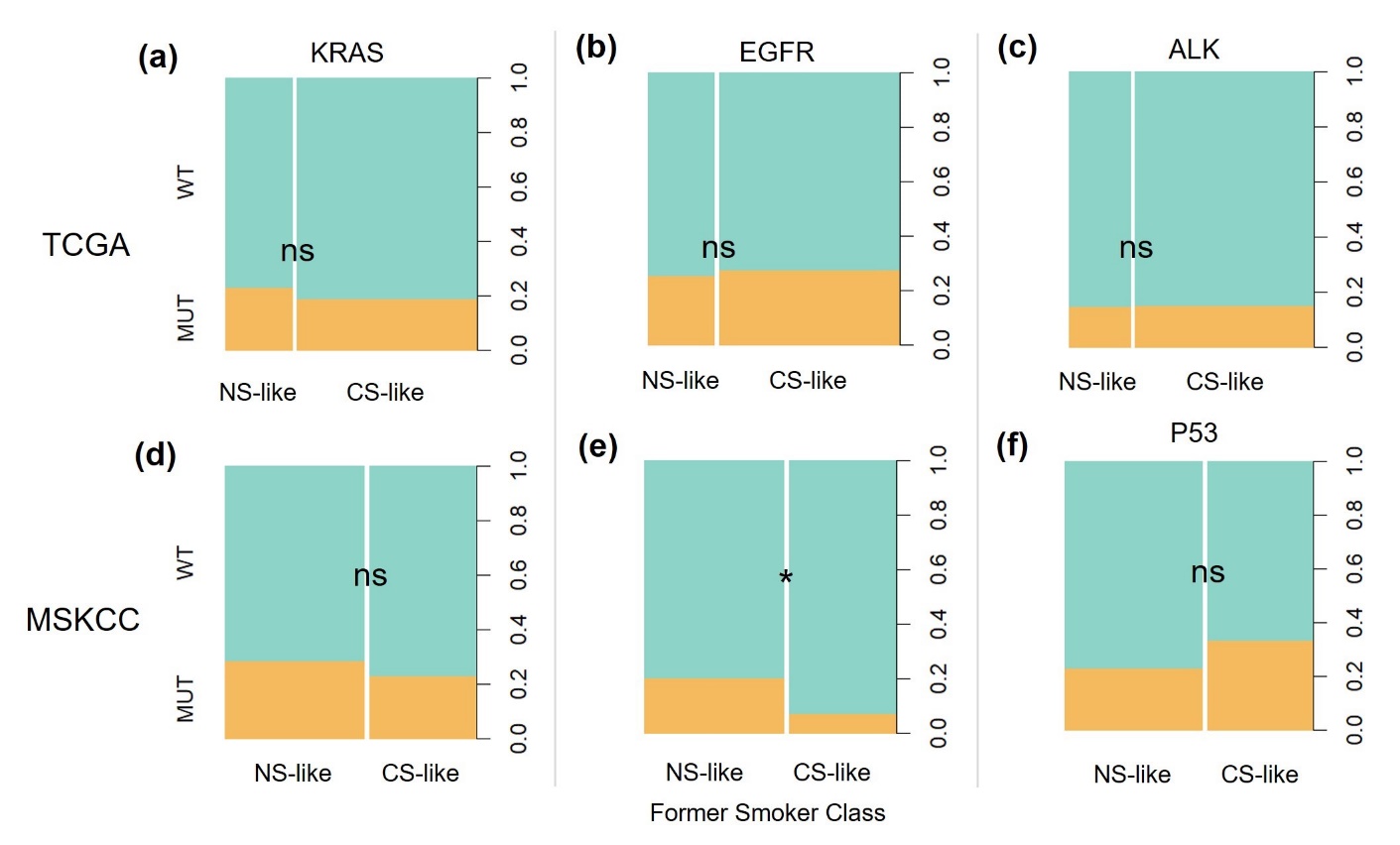


**Figure S4: Proportion of patients who had formerly smoked (FS) with lung adenocarcinoma that harbor oncogenic driver mutations** in (a) *KRAS*, (b) *EGFR* and (c) *ALK* relative to their RF classified CS- or NS-like status in the TCGA cohort and (d) *KRAS*, (e) *EGFR* and (f) tumor suppressor *TP53* in the MSKCC cohort. (Fisher’s exact test, ns= not significant, *p < 0.05) MUT = mutated, WT = wild type


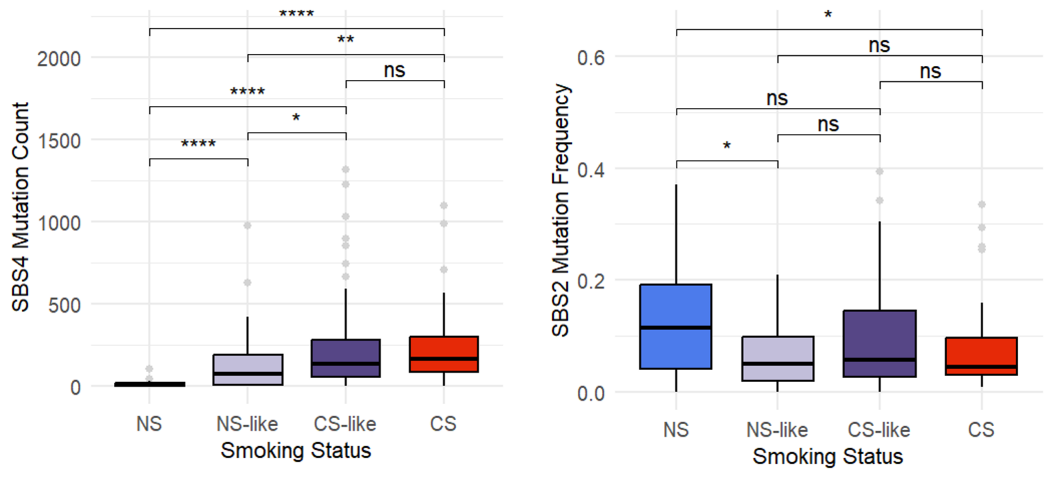


**Figure S5: Absolute levels of tobacco mutagen signature SBS4 and relative mutational signature levels of APOBEC signature SBS2 between different smoking statuses.** ns = not significant, *p < 0.05, **p < 0.01, ****p < 0.0001


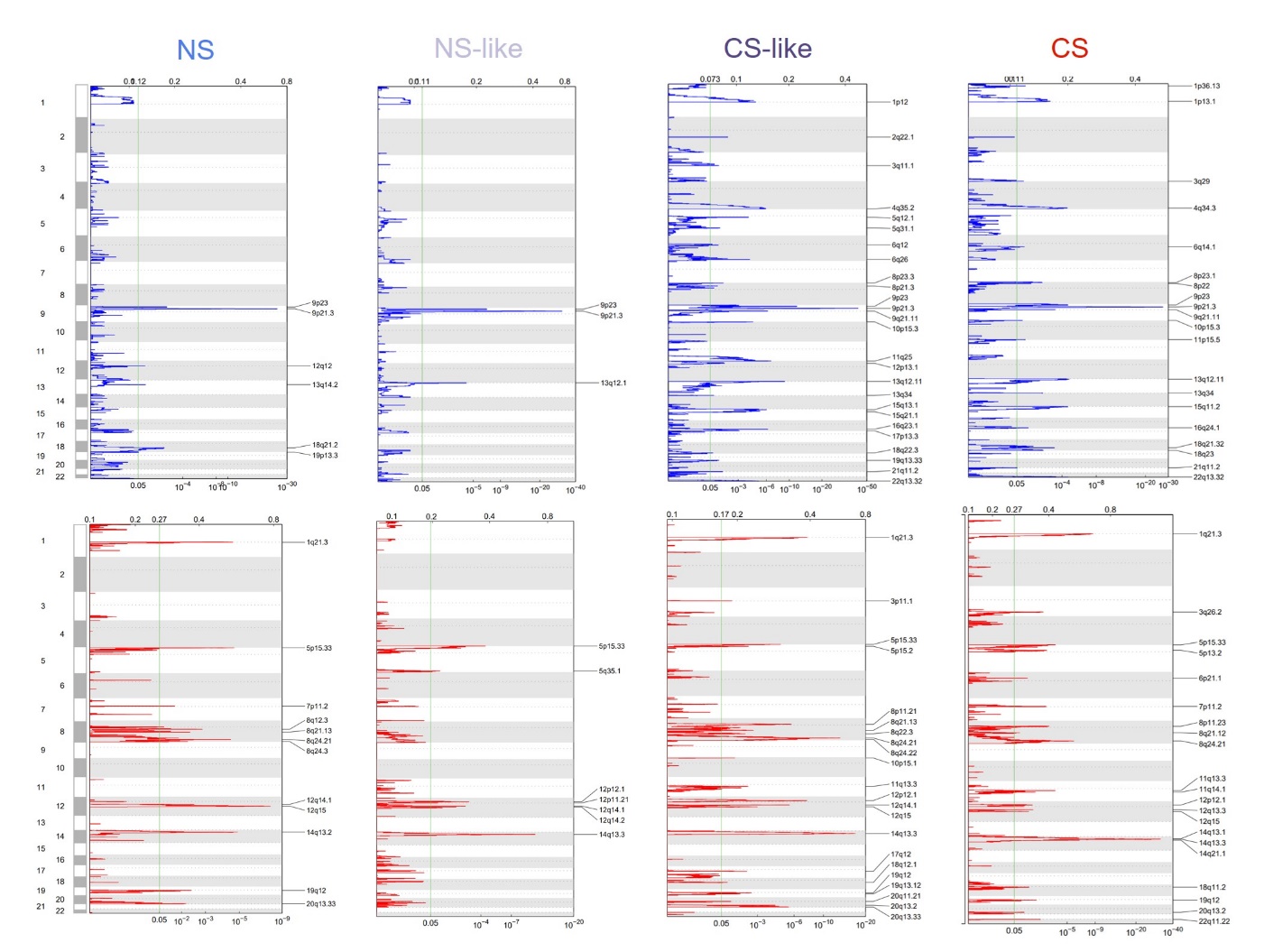


**Figure S6: GISTIC 2.0 analysis of copy number amplifications (red) and deletions (blue) of patients who had never smoked (NS), currently smoke (CS) and NS-like and CS-like patients who had formerly smoked with lung adenocarcinoma patients in the TCGA cohort**. Significance is delineated by a green line at 0.05 representing the false discovery rate corrected p-value.


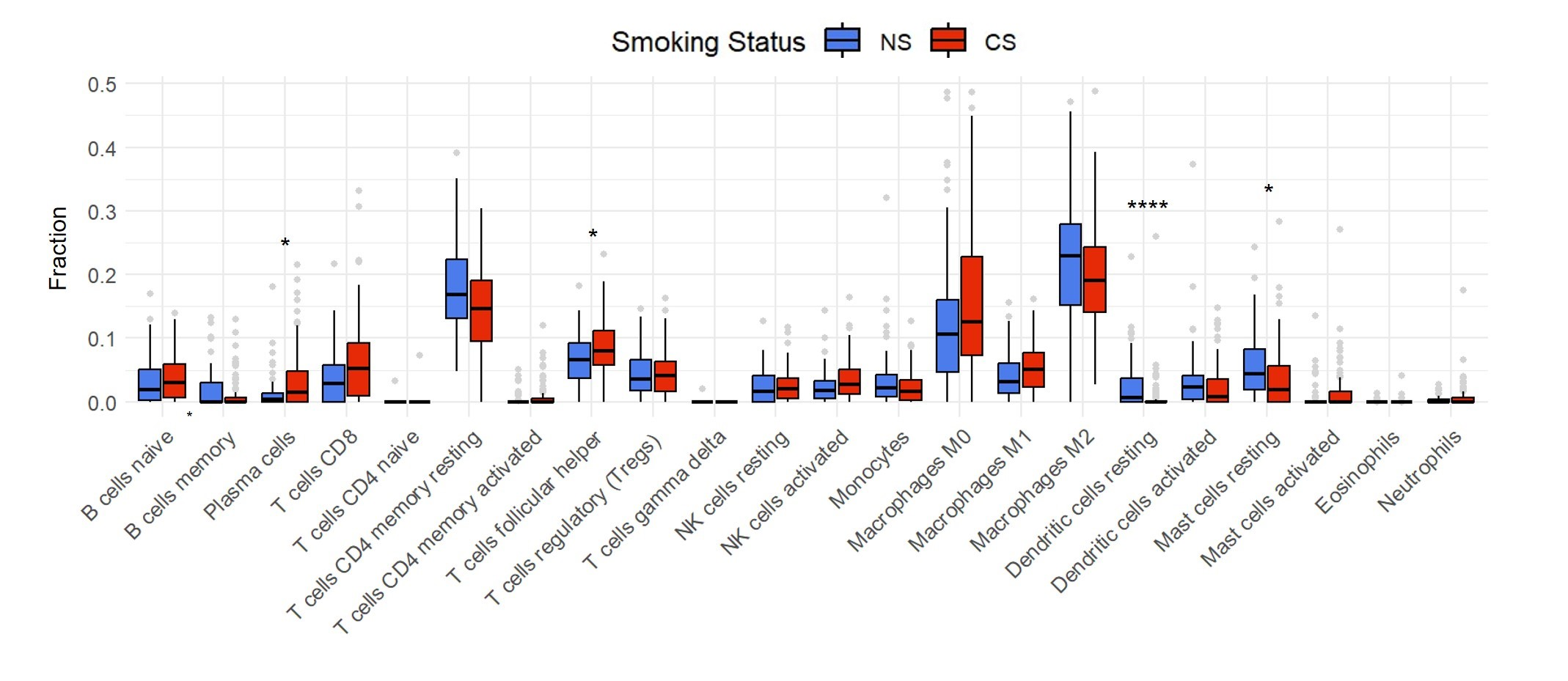


**Figure S7: Comparisons of tumor-infiltrating immune cells between patients who had never smoked (NS) and currently smoke (CS) with lung adenocarcinoma.** (Wilcoxon test and Bonferroni correction, n=193, *p < 0.05, ****p < 0.0001).
