# Supplementary material for: Uncovering molecular features driving lung adenocarcinoma heterogeneity in patients who formerly smoked
Source: J Transl Med. 2024 Jul 8;22:634. doi: 10.1186/s12967-024-05437-8 (PMC11229340; doi:10.1186/s12967-024-05437-8)
Supplement: Supplementary file 2 — Supplementary Material 2 [file 12967_2024_5437_MOESM2_ESM.docx]

**JTRM-D-24-00429 – “Uncovering molecular features driving lung adenocarcinoma heterogeneity in patients who formerly smoked"**

**Response to Reviewers:**

We appreciate the very thoughtful and thorough comments and suggestions provided by the reviewers at Journal of Translational Medicine. We were encouraged that they found our study comprehensive in scope and are enthusiastic about the potential impact of our novel findings. We have addressed all their suggestions as described in the following letter, which we feel substantially improves the paper. Together, we feel these changes now make the paper suitable for publication.

**Reviewer #1:**

***Comment 1-1:*** *The results were illustrated effectively and appropriately. It enables other researchers to easily interpret and possibly build upon the findings, promoting continued exploration in the field.*

**Response 1-1:** We thank the reviewer for taking the time to carefully review our manuscript.

***Comment 1-2:*** *The present study is a well written and well-designed based on public datasets and explore the molecular pathways underline lung cancer focusing on the heterogeneity in lung adenocarcinoma among former smokers. Considering smoking history and study the molecular features-related is a positive and novelty in this study and vital for personalized medicine. By the way, I think it is important to contextualize your findings within the broader landscape of existing research. Are there other studies that have explored similar aspects of lung adenocarcinoma in former smokers? Discussing how your results align with or differ from these studies can provide a deeper understanding of your findings.*

**Response 1-2:** Again, we thank the reviewer for their enthusiasm for our study and agree that contextualizing our results are necessary. To date, there is a lack of studies that explore different molecular aspects of lung adenocarcinoma in former smokers as we have done in our study. This is mainly due to a lack of reporting of former smoking status in previous studies as mentioned in lines 103-113 of the revised manuscript. The studies that currently exist on former smokers that are the most similar to ours are those on reversible and irreversible expression of individual genes after smoking cessation. Our work focuses on the utilization of a collective gene signature to stratify former smoking patients. However, there are overlapping concepts with existing studies and we have now discussed this as requested by the reviewer in lines 276-280 and 408-409 in the discussion section of the revised manuscript.

***Comment 1-3:*** *Why were the Random Forest model chosen over other potential methods? Discussing the advantages of this method in your specific context, as well as considering how the results might differ with alternative approaches, would strengthen the paper. (Do not need to provide the analysis or add it to the manuscript, just explain it to me if possible.)*

**Response 1-3:** Other popular methods to create a model that could classify these tumors include logistic regression, k-nearest neighbours (KNN), and gradient boosting models. The advantages of building a random forest model compared to these other methods is that it is computationally less expensive, is able to handle non-linear data (which is often the nature of gene expression data) and is resistant to overfitting (this is important as our sample size is not very large and overfitting would lead to poor performance on independent test sets and decrease generalizability of our results). However, there is no perfect model as perhaps using a computationally expensive model such as gradient boosting machines may provide more accuracy. Using a KNN model could lead to a choice of k that allows for similar accuracy with less genes required in the model, although KNN is highly sensitive to imbalanced data, and as our groups of current and never smokers were not always of similar size, this would definitely yield different outcomes than using a random forest model.

***Comment 1-4:*** *I recommend using online tools like KM Plotter and https://rnasysu.com/encori/ for additional validation of your prognostic analysis. This can provide a broader context and validation for your results. These additional findings could be provided in a supplementary section (if possible).*

**Response 1-4:** We thank the reviewer for this recommendation. We did look further into these tools and unfortunately KM Plotter only allows an individual gene’s expression to be used for Kaplan-Meier plots and our work focuses on utilizing a collective signature of 123 genes. We also feel that narrowing down our gene signature from 123 is beyond the scope of this study, which is to broadly demonstrate that formerly smoking patients can be meaningfully divided by gene expression. The same is unfortunately true with ENCORI, whose interface only allows a single gene to be chosen for prognostic analysis.

***Comment 1-5:*** *The use of survminer and survival packages for survival analysis is appropriate, but please elucidate why these particular packages were chosen over others. Discussing their specific benefits in analyzing your data can enhance the methodological clarity (Just add one sentence for clarifying).*

**Response 1-5:** The survival package is widely used and cited for survival analyses from gene expression signatures and also in clinical and other molecular contexts due to its extensive documentation and possible integration with other packages. Survminer was developed to allow for customizable and advanced visualizations of survival analysis results, which is why it was used for constructing the figures from the survival model created from the survival package. We have added this clarification in line 220-223.

***Comment 1-6****: Questions on Specific Methods and Analyses:*

a) *Could you elaborate on the use of CIBERSORTx in the immune cell content assessment? How does this deconvolution algorithm specifically aid in your analysis, and what are the implications of its use in your findings?*

**Response:** The CIBERSORTx algorithm uses the gene expression of ~550 genes to predict levels of immune cells present in a bulk tissue sample. This is of interest to us as immune cells have been shown to be prognostic of overall survival and predictive of immunotherapy response. We have mentioned this in lines 205-209 and the implications of the immune cell data have been described in lines 409-412 and 443-447.

b) *In the copy number assessment and mutational analysis part, what were the criteria for choosing the methods and tools used? How do these choices impact the interpretation of your results?*

**Response:** These tools, GISTIC 2.0 and the mutSignatures package are well validated and have been previously cited. GISTIC 2.0 focuses on recurrent events across samples while calculating q-values to control for false positives, other softwares accomplish slightly different goals such as focusing on methylation driven CNAs for CONUMEE or manipulating CNA data instead of identifying significant regions in CNTools. mutSignatures uses standard negative matrix factorization (NMF) method to calculate mutational signatures, which was our main criteria for deciding on its use, but different default parameter settings may lead to slightly different signatures selected and different magnitudes of each signature in other packages like sigProfiler or deconstructSigs.

c) *Regarding the Functional Analysis of Differentially Expressed Genes (DEGs) and Gene Ontology (GO) annotations, can you provide more details on the selection process of these genes and the relevance of the chosen GO terms in the context of your study?*

**Response:** Selection of the DEGs to form the 123 gene signature is delineated in lines 139-146 and also visually represented in Figure 1A. These genes were then all inputted into the DAVID tool and ShinyGO to show which pathways and GO terms were enriched from the 123 genes. This was visualized in Figure 1F and 1G. The relevance of this functional analysis is that it tells us the pathways involved in differentiating current and never smokers and provides context to what is driving our model. These terms and pathways are further detailed in lines 417-425.

***Comment 1-7:*** *It appears that the manuscript does not explicitly discuss the limitations and weaknesses of the study. Addressing limitations is crucial for a comprehensive understanding of the research's scope and the generalizability of its findings and might be helpful for future studies in this field. Here are a few points that might be worth considering:*

a) *Dataset Limitations: If specific datasets were used (e.g., TCGA, BCCA, MSKCC), it would be important to discuss any inherent limitations in these datasets. This might include sample size, diversity of the patient population, or any biases that could impact the findings.*

**Response:** The reviewer brings up an important point and this study has weaknesses in terms of limited sample size and available validation datasets. In addition, all test and validation sets were from North American populations, and from the patients whose race-related data was provided, they were primarily Caucasian. This may limit the generalizability of our results and calls for wider availability of genomic datasets from patients of diverse backgrounds that have reported detailed smoking history. These limitations have now been mentioned in lines 463-465 and 470-474.

b) *Methodological Limitations: While the use of Random Forest models and other statistical methods are innovative, discussing potential limitations or biases of these methods would strengthen the paper. For example, how might the choice of model affect the interpretation of the data?*

**Response:** Random forest (RF) is just one method to create a classifier for current smoker/never smoker status. While it is a relatively robust model, other models may generate coefficients for each covariate, which is not the case for RFs and makes each gene’s magnitude of impact and direction less interpretable and instead require feature importance plots to designate relations of each gene to the data.

c) *Generalizability of Findings: Are the findings specific to the datasets and methods used, or can they be generalized to a broader population? Discussing the extent to which the results can be applied to different populations of lung adenocarcinoma patients would be valuable.*

**Response:** The generalizability of these findings, particularly to different geographical populations is of great interest. We were able to verify our gene signature’s prognostic value in the MSKCC dataset, which is independent from the variable selection and random forest model training process, but that population is predominantly Caucasian. Furthermore, only the TCGA dataset had genomic data available, limiting our conclusions about tumor mutational burden and mutational signatures to one dataset. These limitations have now been mentioned in lines 460-474.

d) *Future Research Directions: Often, limitations can lead to suggestions for future research. Identifying areas that were not addressed in your study but could be explored in future research can provide a roadmap for continuing advancements in this field.*

**Response:** We highlighted future directions stemming off the above comments in lines 466-470, 472-474 and 483-485. We also added other future directions such as investigating this gene signature in normal tissue as well as longitudinally to understand the dynamics of smoking-related gene expression in healthy people over time. These are all encapsulated in lines 475-485.

**Reviewer #2:**

***Comment 2-1:*** *The authors used random forest models and gene expression analyses from lung adenocarcinomas (LUAD) that included patients who were current (CS), former (FS) or never smokers (NS). They developed a signature from 123 genes that classified former smokers into sets of CS-like and NS-like. They examined clinical characteristics and mutational burden, mutagen number and signatures as well as Cibersort to identify immune cell populations. The study is quite comprehensive, and the authors provide extensive data analyses and supplementary information. In general, the study is nicely presented and the main findings that the FS population is heterogeneous is not surprising. A higher mutational burden among FS than NS is also expected. The most interesting finding was CS-like FS tumors had more activated CD4 memory T cells whereas NS-like FS tumors had more resting CD4 memory T cells, monocytes, dendritic and mast cells. A potential analyses that could confirm these findings, would be multiplex IHC to examine the proximity and potential interaction of tumor and immune cell populations. The authors findings that CS-like FS have persistent modified gene expression suggests that methylation or some other mechanism may underlie this effect but was unfortunately not examined. These approaches are likely beyond the scope of the present study.*

**Response 2-1**: We thank the reviewer for taking the time to review our manuscript and for their enthusiasm for our results. These are great insights and we have added this into future directions of the study in lines 468-469.

***Comment 2-2:*** *The authors include limitations but might consider adding the fact that the gene expression and mutational studies all involve sampling of only a small part of a tumor and then the homogenization of the tumor and all of its comprising cells with the loss of the cell to cell context. It is possible that some of the heterogeneity could be understood better if this was more systematically examined.*

**Response 2-2:** This is completely true, and we have added this to our limitations section as well in lines 466-468.
